# Synergistic Effect of Supercritical and Ultrasound-Assisted Ginger (*Zingiber officinale* Roscoe) Extracts

**DOI:** 10.3390/plants11212872

**Published:** 2022-10-27

**Authors:** Taja Žitek, Nika Kučuk, Vesna Postružnik, Maja Leitgeb, Željko Knez, Mateja Primožič, Maša Knez Marevci

**Affiliations:** 1 Faculty of Chemistry and Chemical Engineering, University of Maribor, 2000 Maribor, Slovenia; 2 Faculty of Medicine, University of Maribor, 2000 Maribor, Slovenia

**Keywords:** ginger, supercritical fluid extraction, ultrasound-assisted extraction, anticancer activity, antimicrobial activity

## Abstract

Proper processing of natural material is crucial to obtain an extract with high content of biologically active components. Dried, grinded ginger roots were extracted by ultrasonic method and supercritical extraction with CO_2_. The aim of the study was to determine if a mixture of the two types of extracts attained by different methods and solvents exhibits better bioavailability than each extract itself. Therefore, both extracts were analytically evaluated and then mixed in a ratio of 1:1. The supercritical extract (SCG extract) and the mixed extract (mixG extract) had high antioxidant activity (78% and 73%) and total phenols (827 mg/g ext. and 1455 mg/g ext.), which is also consistent with the levels of gingerol (303 mg/g ext. and 271 g/g ext.) and shogaol (111 mg/g ext. and 100 g/g ext.) in the extracts. In comparison to both pure extracts higher levels of total phenols were found in the extract mixG. This could be the reason for the significant inhibition of melanoma cells and antimicrobial potential (against *Staphylococcus aureus*, *Escherichia coli,* and *Candida albicans)*. The combination of the extracts resulted in a significant increase in the inhibition of selected microbial and melanoma cells WM-266-4 compared to the control. Cell viability decreased below 60% when mixG extract was applied. Antimicrobial activity has been confirmed.

## 1. Introduction

**Figure 1:**
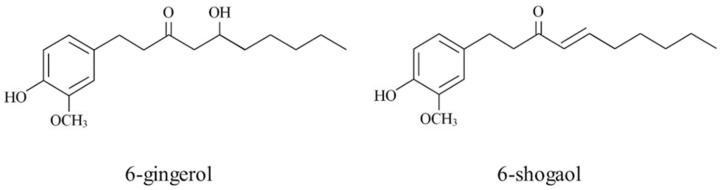
Chemical structure of the two most abundant non-volatile phenolic compounds (6-gingerol and 6-shogaol) in ginger.

Biologically active compounds found in ginger include bioactive phenols, non-volatile compounds with a pungent odor, including gingerols, shogaols, paradols, and zingerones [1]. It also contains terpenic compounds [2]. Among the phenolic compounds, the fresh ginger rhizome contains mainly gingerols, to the greatest extent 6-gingerol, 8-gingerol, and 10-gingerol. Gingerols can be transformed into shogaols, which can be achieved by heat treatment or long-term storage. [1,3,4]. Chemical structures of non-volatile phenolic compounds 6-gingerol and 6-shogaol are shown in Figure 1. Many other phenolic compounds present in ginger, include quercetin, zingerone, gingerenone-A, and 6-dehydrogingerdione [3,5,6].

6-Gingerol is the major phenolic compound derived from ginger and has antibacterial, anti-inflammatory, and anti-tumor activity. While several molecular mechanisms have been described to underlie its effects on cells in vitro and in vivo, the underlying mechanisms by which 6-gingerol exerts anti-tumorigenic effects are largely unknown. Huang et al. indicate that 6-gingerol inhibits melanogenesis of B16F10 melanoma and can function as a good skin whitening agent [7]. Park et al. suggest that 6-gingerol can circumvent the resistance of mutant p53-expressing cells towards chemotherapy by inducing apoptotic cell death while it exerts a cytostatic effect on wild-type p53-expressing cells by inducing temporal growth arrest [8]. 

In addition to exhibiting exceptional anticancer activity, ginger has also shown remarkable potential in inhibiting the growth of pathogenic microorganisms, including bacteria and fungi [3,9,10,11]. 

Excessive use of antibiotics has increased resistance to pathogenic microorganisms, which poses a significant growing challenge in the successful treatment of serious infectious diseases. Therefore, increasing attention is being paid to finding new antimicrobials of natural origin, such as ginger and its active ingredients. In general, Gram-negative bacteria are more resistant to various antibiotic agents due to the presence of an outer membrane. While Gram-positive bacteria are without this outer layer but have a thicker layer of peptidoglycan and antibiotics can inhibit their growth more effectively [12]. Bioactive compounds from ginger have the ability to affect the integrity of the microbial cells membrane as they can cause permeability of the cell wall and cytoplasmic membrane [3]. On the other hand, yeasts such as *Candida albicans* can be highly adaptable microorganisms, as they can develop resistance after prolonged exposure to antimicrobial agents [13].

The antimicrobial efficacy of ginger extracts and proportion of essential oils depends mainly on the extraction technique used, the extraction solvent, and the consequent chemical composition [14,15,16]. Ginger extracts generally show better inhibitory properties on the growth of Gram-positive than Gram-negative bacteria [17]. *Staphylococcus aureus* and *Escherichia coli* have been shown to be sensitive to ethanolic ginger extract [18,19,20]. Excellent inhibitory properties on the growth of the yeast *C. albicans* were also detected [21,22,23,24]. Among others, ginger extracts and essential oils also showed potential antimicrobial activity on the growth of some other bacteria, including *Bacillus cereus*, *Listeria monocytogenes*, *Klebsiella pneumoniae*, *Bacillus subtilis*, *Pseudomonas aeruginosa*, *Pseudomonas stutzeri*, *Salmonella typhimurium*, and fungi, such as *Aspergillus niger*, *Aspergillus flavus*, and *Penicillium* spp. [14,25,26]. The main bioactive substances that significantly contribute to the antimicrobial activity of ginger are 6-gingerol and 6-shogaol [27]. 

The effect of plant extracts such as turmeric, goji berries, horsetail, grape and rosemary on the metabolic activity of melanoma cells (WM-266-4) was studied in our previous studies [28]. We also performed an analysis of the effect of a mixture of cannabis and ginger extract in one of our studies [29], with a sample concentration of 1 mg/mL being the marginal concentration that is required for inhibition of further cancer cell division. However, in the reviewed literature, we did not find that these analyses were performed on the mentioned cell line in any other study. Therefore, this is the first study to analyse the effect of ginger extracts obtained by two different extraction techniques ultrasound-assisted extraction (UAE) with ethanol and supercritical fluid extraction (SFE) with carbon dioxide, as well as their mixture, on the metabolic activities of melanoma cells (WM-266-4).

The aim of the present research was to evaluate and characterize the mixture of the commercial ginger extracts obtained by two different extraction methods, the UAE and the SFE, as described in our previous study [29], by determining antioxidant activity, total phenolic content, and the content of two important bioactive compounds 6-gingerol and 6-shogaol. Compared to the reviewed literature, in our study, a mixture of extracts obtained by two different extraction procedures with solvents was characterized for the first time in order to achieve a synergistic effect of various bioactive compounds, which could further contribute to improved absorption of these substances by oral administration.

Two separate extracts have been prepared from the dried ground ginger; UAE and SFE have been carried out and each extract and their mixture have been characterized by determining antioxidant activity and total phenolic content. The qualitative and quantitative analyses of ginger components were performed by liquid chromatography coupled with mass spectrometry. In addition, antimicrobial (Gram-positive bacteria *S. aureus*, Gram-negative bacteria *E. coli*, and yeast *C. albicans*) and anticancer activities were investigated. The antimicrobial activity was performed by the broth dilution method, and the anticancer activity was tested on WM-266-4 melanoma cells.

The obtained extracts were further mixed in a ratio of 1:1. A combination of the extraction methods was selected with the aim to isolate the full spectrum of ginger compounds, both polar and nonpolar bioactive substances, which has not been presented in any study so far. Polar and nonpolar compounds cannot be mixed; however, in our study, we managed to prepare a stabile homogenous mixture of extracts with proper preparation, heating, and use of an emulsifier. Therefore, a combination of obtained extracts was used for further analysis due to the synergism between different polar and nonpolar compounds obtained by different extraction methods. 

## 2. Results and Discussion

In this study, characterization of UAG (extract obtained by ultrasound-assisted extraction) and SCG (extract obtained by supercritical fluid extraction) extracts derived from a mixture of extracts obtained by UAE and SFE was used. Their combination was performed to evaluate a synergistic effect of bioactive compounds. This would significantly contribute to the improvement of biological activity, as well as enhance absorption during oral administration, determined by studying the crystalline profile of the extracts. Therefore, the antioxidant, antimicrobial and anticancer activity of ginger extracts was evaluated. The content of two important bioactive components 6-gingerol and 6-shogaol, and the content of total phenols, expressed in gallic acid equivalent, were also determined.

### 2.1. Extraction Yield and Crystallinity Profile

In order to improve the biological activity, the content of biologically active compounds, as well as antimicrobial and anticancer activity, a mixture of extracts obtained by two different extraction techniques was prepared. UAE and SFE of dried ginger were performed at selected optimal conditions determined in our preliminary study [29]. In the UAE method, ethanol, as a solvent with a relative high polarity, was used to yield polar substances from dried ginger. In comparison, SFE was performed with a nonpolar solvent, namely supercritical CO_2_ (SC CO_2_), which consequently led to the extraction of primarily nonpolar active ingredients. The extraction process using ultrasound successfully disrupts the cell wall, causing the polar substances of the cell wall to dissolve in ethanol, a polar solvent. On the other hand, nonpolar compounds are efficiently extracted using SFE.

Extractions were repeated four times with average yield values given with both methods. Depending on the material, the yield of the extract was slightly higher in the case of UAE (*η* = 6.37 ± 0.02%, conditions: room temperature, 30 min, ethanol) than in the case of SFE extraction (*η* = 4.58 ± 0.10%, conditions: 60 °C, 300 bar, 3 h, CO_2_). Our results are in agreement with the available literature data. Mesomo et al. [30] achieved comparable yields (*η* = 3.21%) in their study under similar SFE conditions (60 °C and 250 bar) as in our study (60 °C and 300 bar). Fitriady et al. [31] determined the SFE yields under different operating conditions, where the maximum yield (2.9%) for a four-hour extraction was achieved at 40 °C and 310 bar. Shukla et al. [32] also optimized SFE conditions to achieve the maximum yield, where at the same time, the highest amount of non-volatile oil components (6-, 8-, 10-gingerols and 6-shogaol) was obtained. Optimal extraction conditions for the highest achieved yield of 8.6% were 40 °C and 276 bar at an extraction time of 153 min. In a study by Supardan et al. [33], the extraction efficiency increased with prolonged ultrasound exposure of ginger in ethanol. Under similar conditions, the yield of UAE (*η* = 5.50%) coincides with the yield achieved in our study. Depending on the choice of extraction technique and the solvent used, the content of isolated bioactive substances from ginger and other properties also differed.

**Figure 2:**
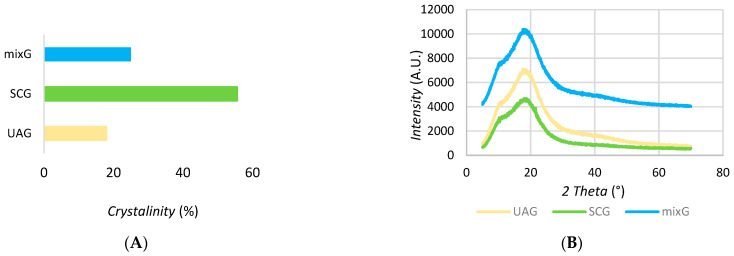
(**A**) The columns show the crystallinity profile. (**B**) XRD patterns of tree extracts showing *intensity* (A.U.) with respect to *2 Theta* (°).

The crystallinity profiles of the extracts were studied by X-ray diffraction. Figure 2 shows the X-ray diffractograms for UAG extract, SCG extract, and mixG extract: graph B show the dependence of Intensity (A.U.) according to 2 Theta (°). When comparing the diffraction patterns of the different extracts obtained, a decrease in peak intensity was observed for UAG and mixG extract, indicating a lower crystallinity and thus a potential increase in biodegradability. This also indicates that SC CO_2_ more selectively isolated non-amorphous components (visible, for example, in the range of 30 to 50). The results are important for further studies in formulations, exploring the release of medicinal components in the body. 

The shape of the diagram in Figure 2 represents an amorphous profile in which the building blocks are disordered, as opposed to crystalline substances, which are characterized by the correct arrangement of the building blocks. When amorphous compounds are taken orally, the water molecules have easier access to the biologically active components and consequently better absorption of the active ingredients in the human body.

### 2.2. Characterization of Extracts Using LC-MS/MS Method

The two most important non-volatile bioactive compounds present in ginger, 6-gingerol and 6-shogaol, were determined by the LC-MS/MS method for extracts obtained by two different extraction techniques, including their mixture. Table 1 shows their concentration, expressed as mg of each compound per g of extract. Both compounds make an important contribution to the antimicrobial properties of the ginger extract, as well as to the anticancer effect.

As seen in Table 1, SCG extract contained almost two times higher concentration of both components (302.49 ± 4.63 mg/g extract of 6-gingerol and 110.96 ± 6.64 mg/g extract of 6-shogaol) than UAG extract. On this basis the mixG extract contained a high concentration of both components (270.63 ± 2.04 mg/g extract of 6-gingerol and 99.76 ± 0.87 mg/g extract of 6-shogaol), as well. In all extracts the content of 6-gingerol was higher than 6-shoagol. To the best of our knowledge, no study has been published to determine the content of these compounds in a mixture of UAG and SCG extracts as in this study. However, the results of 6-gingerol and 6-shogaol content in UAG and SCG extracts alone are comparable to those achieved in the literature. Sonale et al. [34] also demonstrated a higher content of 6-gingerol than 6-shogall in their SCG extracts. In contrast, Foudah et al. [35] achieved much lower concentrations in UAG extracts obtained from various ginger starting materials compared to our results, and higher content of 6-shogaol than 6-gingerol. The content of 6-shogaol was between 10.7 ± 0.4 and 19.7 ± 1.0 mg/g extract and the content of 6-gingerol between 0.2 ± 0.6 and 15.1 ± 0.8 mg/g extract. Under different operating conditions of SFE (10–15 MPa and 35–45 °C), Salea et al. [36] achieved comparable content of 6-gingerol. Compared to the other extraction processes used, namely high-pressure Soxhlet with liquid CO_2_, Soxhlet with n-hexane, and percolation with ethanol (96% *v/v*), the highest content of 6-gingerol was obtained in the SCG extract. The highest 6-gingerol content by SFE (20.7%) was achieved at 15 MPa and 35 °C, and when Soxhlet extraction with n-hexane or ethanol was used, low 6-gingerol content (4.59–6.26%) was isolated. For extracting polyphenols from ginger, ethanol has proven to be the most suitable due to its higher polarity than acetone and methanol [37]. In addition, the main disadvantage of conventional methods is the residual solvent. Furthermore, high temperatures significantly influence the extraction yield and the chemical composition of ginger extracts [4,38].

The main non-volatile phenolic compounds of ginger, 6-gingerol and 6-shogaol, significantly contribute to antimicrobial and anticancer activity. Due to the high content of both active ingredients in all three types of ginger extracts, the extracts should expose high antimicrobial as well as anticancer properties, as presented in the following subsections.

### 2.3. Antioxidant Activity and Total Phenolic Content of Ginger Extracts

Extracts with different content of active ingredients can be obtained by SFE or UAE, mainly due to the different polarity of the solvent used. Therefore, the total phenolic content and antioxidant potential of ginger extracts with the above mentioned extraction techniques were studied. In order to verify the synergistic effect of polar and nonpolar biologically active compounds, we also analyzed the combination of both extracts. The spectrophotometric methods were used for the determination of the content of these important bioactive substances and antioxidant activity. Obtained results are presented in Table 2. The total phenolic content of the extracts was measured using the Folin–Ciocalteu reagent, and the results were expressed as mg of gallic acid equivalents per 100 g of extract (mg GAE/100 g). The antioxidant activity was determined by the DPPH assay, expressed as % inhibition. The highest level of total phenols was determined in the combined extract (1455.37 mg/100 g). It is assumed that a synergistic effect occurred between the nonpolar components of the SCG extract and the polar components of the UAG extract which exhibited a high content of total phenols (1342.59 mg/100 g). On the contrary, antioxidant activity did not reveal major differences between the extracts. However, the highest values were measured for the SCG extract (78.17%).

As can be seen from Table 2, the mixture, mixG extract achieved a higher content of concentration of total phenols than each individual extract. In contrast, the antioxidant activity of the mixG extract reached a mean value between both extracts. The highest radical scavenging activity was detected in the SCG extract. One way ANOVA showed significant differences between extraction methods for extract antioxidant activity (F = 69.31, *p* < 0.001 and phenolic content (F = 393.6, *p* < 0.001). While post hoc Tuckey test showed significant differences between all extraction methods for antioxidant activity and phenolic content (*p* < 0.001). The Pearson correlation test showed a strong correlation between antioxidant activity of the extracts and 6-gingerol (r = 0.935, *p* < 0.001) as well as for 6-shoagol (r = 0.948, *p* < 0.001). This confirms 6-gingerol and 6-shoagol as two of the main components in ginger responsible for its antioxidant activity.

Comparing single extracts, the higher total phenolic content was measured in the case of UAG extract, where mainly polar phenolic compounds were isolated, due to the polar nature of the solvent used. As opposed to SCG extract, primarily nonpolar phenolic components were obtained. While the antioxidant potential was otherwise better in the case of SCG extract, the % inhibition differed from the UAG extract by only about 8.4%. 

Therefore, it can be concluded that both obtained ginger extracts, including their mixtures, are a rich source of phenolic compounds with high antioxidant activity. The excellent results obtained for the mixture of UAG and SCG extracts are the main reason that further analyses were carried out only for the mixture due to the potential synergistic effects of polar and nonpolar active substances.

The results of the total phenolic content in the UAG ethanol extract of ginger are in accordance with the literature [39]. Regarding the antioxidant potential, it is also in line with our results, as Stoilova et al. [40] achieved comparable results for the SCG extract, with a maximum radical scavenging activity of 90.1%. Compared to the obtained results in the study of Murhphy et al. [41], our extract showed better antioxidant properties, namely a 1.3-fold higher percentage of inhibition for UAG extract. Using different operating conditions of SFE (30–50 °C, 138–276 bar), Rehman et al. [42] achieved in their study much lower total phenolic contents in ginger extract (312.44 ± 1.94–456.11 ± 10.19 mg GAE/100 g) compared to the results of our research. Additionally, with different operating conditions of UAE (ethanol/methanol, 30–50 °C, 20–40 min), lower total phenolic content was detected (200.33 ± 1.45–300.12 ± 7.01 mg GAE/100 g). In contrast, Jaiswal and Nail [43] measured comparable values of antioxidant activity in the SCG extract but slightly higher values of total phenols, 2110 ± 0.62 mg GAE/100 g. In comparison, the measured concentration in Soxhlet extract was even higher, 3850 ± 0.96 mg GAE/100 g. Similar results were achieved by Ezez and Tefera [44] using different extraction solvents, namely ethanol, methanol acetone, and ethyl acetate. Total phenolic content ranged between 748.865 ± 0.210 and 1183.813 ± 0.418 mg GAE/100 g, and DPPH radical scavenging activity between 70,597 ± 0.332 and 84,868 ± 0.293% inhibition. However, Chan et al. [11] detected only 157 mg GAE/100 g in the methanol extract, obtained with conventional swirling using an orbital shaker.

For the first time, studies of bioactive compounds content for a mixture of ginger extracts obtained by different extraction methods were performed. With our study we can confirm that the use of a combination of extracts significantly contributes to better biological activity, due to the possible synergistic effect of polar and non-polar compounds.

### 2.4. Antimicrobial Activity of Extract Mixture

Due to the growing resistance of pathogenic microorganisms to antibiotics and other antimicrobial agents, more attention is paid to the search for new alternative antimicrobials of natural origin. For this reason, the antimicrobial efficacy of a mixture of UAG and SCG extract against the growth of selected pathogenic microorganisms, Gram-positive bacteria *S. aureus*, Gram-negative bacteria *E. coli*, and yeast *C. albicans,* which can cause various infections, were studied. 

The inhibitory properties of mixG extract were quantified by the microbroth dilution method, where the minimum inhibitory concentrations (MICs) were determined. The MIC is the minimum sample concentration at which inhibition of growth of the test microorganism is successfully achieved. The MIC values for mixG extract against the abovementioned two bacterial species *E. coli* and *S. aureus* and yeast *C. albicans* are presented in Table 3. MixG extract most effectively inhibited the growth of yeast *C. albicans* at a sample concentration of 40 mg/mL. The lowest inhibitory concentration of mixG extract was 60 mg/mL for *E. coli* and 70 mg/mL for *S. aureus*. In this regard, *S. aureus* proved to be the most resistant among the microorganisms tested.

All tested microorganisms were sensitive to active compounds from the tested mixture of ginger extracts. The best inhibitory properties of the extract were detected in the case of the yeast *C. albicans*, as seen in Table 3, which can be attributed to the high content of 6-gingerol (270.63 mg/g extract). 

Olannye et al. [45] concluded that *C. albicans* was resistant to a ginger extract obtained by the Soxhlet extraction method with methanol at a concentration of 100 mg/mL, while in our study, it was the most sensitive among the microorganisms tested. A wide range of MIC values of the extracts against the observed microorganisms is reported in the literature. Panpatil et al. [46] determined higher MIC values of aqueous and ethanolic extracts against *E. coli* and *S. aureus*, namely, 175 mg/mL and 125 mg/mL, respectively. To determine the antimicrobial potential of ginger, Yassen et al. [47] prepared different extracts using water and various organic solvents such as ethanol, methanol, hexane, acetone, and chloroform. MIC values of 62.5 mg/mL were reported for the ethanol extract against the two bacteria tested, *E. coli* and *S. aureus*. Our results of mixG extract are comparable for both bacterial species tested. 

In general, Gram-negative bacteria are considered to be more resistant to various antimicrobial agents due to the presence of the outer membrane than in contrast to Gram-positive bacteria. Using a mixture of ginger extracts obtained by two different extraction techniques UAE and SFE, we demonstrated that the Gram-negative bacteria *E. coli* was more susceptible than the Gram-positive bacteria *S. aureus*. On the other hand, most yeasts are more adaptable, so they can develop antimicrobial resistance faster than bacteria. However, in our study, we proved that the mixture of extracts most inhibited the growth of the tested yeast *C. albicans*.

### 2.5. Effect of Ginger Extract on Metabolic Activity of Metastatic Cells 

**Figure 3:**
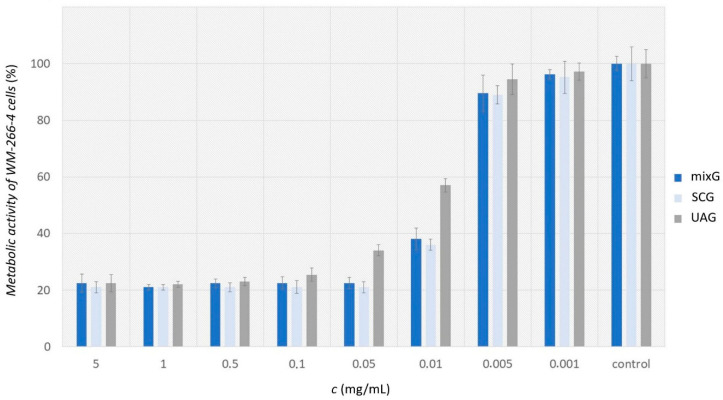
Metabolic activity of metastatic cells WM-266-4 after applying the extract of selected concentrations.

MixG, SCG and UAG extracts were applied to metastatic cells WM-266-4. The percentage of metabolic activity of the cells after applying the extract was determined in comparison to the control, representing 100% cell function. The activity of metastatic cells significantly decreased at a concentration of the applied extracts solution of 0.01 mg/mL in Figure 3 (MA_mixG_ ≐ 38%, MA_SCG_ ≐ 36% and MA_UAG_ ≐ 57%). 

Figure 3 shows that the highest decrease in metabolic activity of cells was obtained with the SCG extract. However, there was no significant difference between the extracts mixG and SCG in terms of deviation values. While the UAG extract had a slightly lower effect on reducing the metabolic activity of cancer cells. From the measurements and cell morphology, it is evident that the concentration of 0.05 mg/mL completely inhibited the activity of cancer cells when the extracts mixG and SCG were applied. Inhibition also occurred when UAG extract was applied, but a higher concentration (0.1 mg/mL) was required.

**Figure 4:**
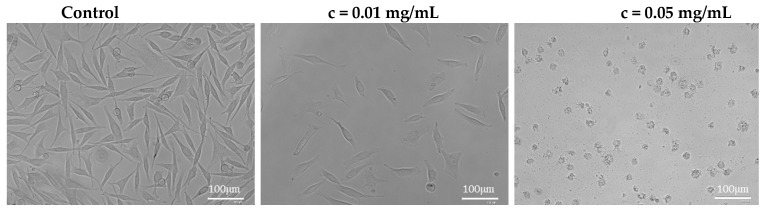
Morphology of melanoma cells WM-266-4 after applying the mixG extract solution concentrations 0.01 mg/mL and 0.05 mg/mL compared to control.

Figure 4 shows the morphology of melanoma cells WM-266-4 after application of the mixG extract solution at concentrations of 0.01 mg/mL and 0.05 mg/mL compared with the control. At this concentration, the correct shape of cells could be observed despite their small numbers (Figure 4). On the other hand, when the extract solution was applied at a concentration of 0.05 mg/mL, the cells changed their shape completely. The metabolic activity of the cells at this concentration was only 22.5%. The results show significant inhibition of further division of cancer cells after application of the combined ginger extract at a concentration of 0.05 mg/mL. Further apoptosis studies would be required to confirm complete cell death.

The significant inhibition of metastatic cell function in this study is thought to be due to the high 6-gingerol content (271 mg/g extract).

## 3. Materials and Methods

### 3.1. Materials

Ginger was obtained from a local Slovenian vendor (Mercator d.d., Ljubljana, Slovenia). The natural material was dried by a lyophilization process (*T*_lyophilization_= −30 °C, *t*_lyophilization_= 72 h, *p* = 0.04 mbar vacuum) and then ground using a grinder before extraction procedures. The mean particle size has been determined by sieve analysis and was 0.5 mm.

Chemicals used in this study were ethanol (C_2_H_6_O) (Sigma-Aldrich, St. Louis, MO, USA, HPLC grade, ≥99.9%), carbon dioxide (CO_2_) (Messer; MG, Ruše, Slovenia, purity 2.5), 2,2-diphenyl-1-picrylhydrazyl (DPPH) (SigmaAldrich, Darmstadt, Germany, ≥97.0%), Folin-Ciocalten reagent (FC) (Sigma Aldrich), sodium(V)carbonate (Na_2_CO_3_) (Sigma Aldrich, ≥99.9%), gallic acid (C7H6O5) (Sigma Aldrich, 97.5–102.5%), and methanol (H_3_OH) (Honeywell, Charlotte, NC, USA, LC-MS CHROMASOLV^®^, ≥99.9%).

### 3.2. Extraction Procedures

Based on preliminary research [29] and the reviewed literature, two different extraction techniques were performed, UAE with ethanol as a solvent and SFE with CO_2_. All extractions and consecutive measurements were performed in triplicate.

#### 3.2.1. Supercritical Fluid Extraction

**Figure 5:**
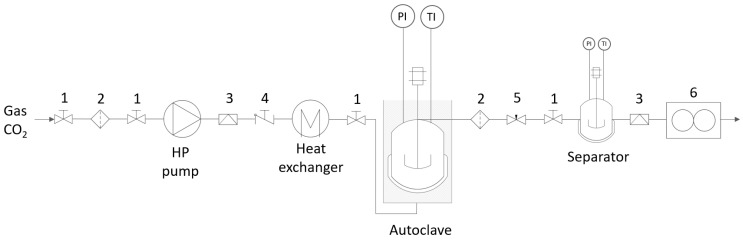
Schematic representation of the SFE process: 1 = valve, 2 = high pressure filter, 3 = rupture disc, 4 = one way valve, 5 = regulating valve and 6 = flowmeter [29].

SFE with CO_2_ was performed in a semi-continuous apparatus (Figure 5), as explained in detail by Kotnik et al. and Žitek et al. [29,48]. Ground ginger (15 g) was put in the autoclave. The liquefied CO_2_ was continuously pumped by a high-pressure pump through a preheating coil into the autoclave. The temperature of the water bath was maintained constant. Separation was carried out in a separator at a pressure of 1 bar. The extract collected into a tube at the bottom of the separator was weighed and the yield was calculated. The extraction was conducted for three hours at selected optimal operating conditions of 60 °C and 300 bar with CO_2_ solvent of feed S/F = 116.67. The extract was stored in a freezer at −20 °C until further analysis.

#### 3.2.2. Ultrasound-Assisted Extraction

The extraction method using ultrasound was used to isolate the polar bioactive compounds from ginger with ethanol. Ground ginger (15 g) was mixed with 150 mL of ethanol in an ultrasound bath at a frequency of 40 kHz. Extraction was performed for 30 minutes at room temperature. The obtained mixture was filtered through filter paper. The residue on the filter paper was discarded, and the solvent was removed from the filtrate using a rotary evaporator under reduced pressure at 40 °C (Büchi^®^ Rotavapor R-144, Flawil, Switzerland). The extract was stored in a freezer at −20 °C until further analysis.

#### 3.2.3. Preparation of Extract Mixture

A mixture of extracts obtained by UAE (UAG extract) and SFE (SCG extract) was used for further analysis. Both extracts, with a higher proportion of polar compounds (UAG extract) and nonpolar compounds (SCG extract), were formulated into a stable mixture with an emulsifier Tween 80. The combined sample of extracts was prepared in a 1:1 ratio (mixG extract). The mixG extract was diluted before further analysis. In the case of the microdilution method, the extract was dissolved in Mueller–Hinton (MH) broth. When the mixG extract was applied to the melanoma cells, it was diluted in DMSO and cell medium. The amount of DMSO in the solution was 0.7%.

### 3.3. Characterization of Extracts

#### 3.3.1. LC-MS/MS Method 

LC-MS/MS analysis was performed using Agilent 1200 HPLC instrument in conjunction with an Agilent 6460 JetStream Triple Quadrupole (QqQ) mass spectrometer. (The column: Phenomenex Kinetex C18; 150 × 2.1 mm ID, 2.6 μm particles). The gas temperature was 150 °C, and the gas flow was 5 L/min. The nebulizer voltage was optimized to 30 V, and the sheath gas temperature was 300 °C with a flow of 11 L/min. The capillary and nozzle voltages were 3500 V and 1000 V, respectively. The optimized gradient program of the mobile phase (A: 0.1% formic acid in water, B: 0.1% formic acid in acetonitrile) was used: 0 min 50% B, 3 min 90% B, 6 min 90% B, 7 min 50% B and held for 5 min. The mobile phase flow was 0.5 mL/min at 30 °C, and 5 µL of the sample was injected onto the column.

#### 3.3.2. Determination of Extracts Biological Activity 

##### Antioxidant Activity

The antioxidant activity of the obtained extracts was determined using the DPPH method described in detail in a previous study [28]. First, sample solutions were prepared in methanol at a concentration of 1 mg/mL. To 77 µL of the sample solution, 3 mL of a prepared methanolic DPPH solution with a concentration of 6 × 10^−5^ M was added. In parallel, the same procedure was used to prepare a blank sample containing methanol instead of a sample. After 15 minutes of incubation in a dark room at room temperature, absorbance was measured at a wavelength of 515 nm. Antioxidant activity was expressed as a percentage of inhibition.

##### Total Phenolic Content

The content of total phenols was determined using the Folin–Ciocalteu method, described in detail by Škerget et al. [49]. First, a solution of FC reagent (Folin–Ciocalteu stock solution diluted 1:10 in distilled water), a solution of NaCO_3_ (75 g/L), and a stock solution of gallic acid at a concentration of 0.4 mg/mL were prepared. 2.5 mL of the FC reagent solution and 2 mL of the NaCO_3_ solution were added to 0.5 mL of distilled water (blank) or a sample with a concentration of 2 mg/mL. Incubation at 50 °C for 5 minutes was followed. The absorbance of the cooled samples was measured at 760 nm. A calibration curve for gallic acid in distilled water was prepared by the same procedure. The results were expressed in grams of GAE per milligram of extract.

##### Antimicrobial Activity

The antimicrobial potential was measured using the microdilution method described in a previous study [29]. Briefly, the prepared emulsions (extract mixG emulsions) were applied to *S. aureus* (MRSA) (ATCC 25923, ATCC, Wesel, Germany), *E. coli* (ATCC 25922, ATCC, Wesel, Germany), and *C. albicans* (ATCC 60193, ATCC, Wesel, Germany). Bacterial growth was confirmed by adding 25 μL sterile 0.04% dye solution of the fluorogenic dye resazurin blue (7-hydroxy-10-oxidophenoxazin-10-yum-3-one, sodium), used as a redox indicator. After four hours of exposure of the resazurin to the microorganisms, the MIC was determined based on the color change. With live bacterial cells, the resazurin turned pink. Columns without discoloration were considered as upper minimum inhibitory concentration (MIC). Ten concentrations of extract mixG solutions (*c* = 60, 50, 45, 40, 35, 30, 25, 20, 15, 5 mg/mL) were applied to the microorganisms. Sterile controls were prepared during the procedure. The positive control contained MH broth and diluted extract mixG, and the negative control contained MH broth and microbial cells. The inoculum density of the added bacteria was 10^8^ colony-forming units per milliliter (CFU/mL), and the volume used was 10 μL.

##### Application of Extract on Metastatic Cells WM-266-4

The human melanoma cell line WM -266-4 (ATCC®, USA) was grown in Eagle’s minimum essential medium (EMEM, ATCC®, USA) containing 10% fetal bovine serum (FBS, Sigma-Aldrich Chemie GmbH) and the antibiotic MycoZapTM (Lonza, Switzerland). Cells were grown in an incubator (Sanyo, Japan) at 37 °C in an atmosphere of 5% CO_2_.

After cell growth, a trypsinization procedure was carried out. HEPES buffer was added to the cells and removed after 10 seconds. Trypsin solution and TNS solution were then added. After centrifugation, the liquid phase was pipetted off, and the remaining cells were diluted with a complete medium. After incubation, the extract mixG solution was applied to the cells, and then the metabolic activity of the cells was determined using the WST 8 assay. CCVK-1 reagent was used, and the amount of formazan formed was measured using a UV-VIS spectrophotometer at 450 nm. The effect of a particular concentration of extract mixG on the metabolic activity of the cells was determined using a control sample in which the cells were exposed to the pure medium.

#### 3.3.3. X-ray Diffraction Spectroscopy

X-ray Diffraction Spectroscopy (XRD) of extracts was recorded on a D2 X-ray diffractometer (Bruker Siemens). Reflections at 2θ were observed between the range 10° and 70°, with an increment of 0.03°, using a Si holder at a voltage of 30 kV and current of 10 mA. The relative crystallinity degree of the extract phase was determined by the ratio between the integrated area of the crystalline peaks and the total integrated area of the diffraction spectra [50].

### 3.4. Statistical Analysis

The differences in antioxidant activity and total phenolic content of the extracts were examined using R programming language version 4.1.1. and RStudio version 1.4.1717. The distribution of data was evaluated using the Shapiro–Wilk statistical test. Obtained data are distributed normally and are presented with mean and standard deviation. Analysis of variance (ANOVA) was performed to compare the content of biologically active components between extraction methods. A post hoc Tuckey honest significance test was performed to evaluate significant differences between groups. The Pearson correlation test was used to examine the relationship between antioxidant activity and the levels of 6-gingerol and 6-shogaol in the samples.

## 4. Conclusions

The profiles of compounds in ginger extracts obtained by two different extraction techniques, UAE using ethanol as a solvent and SFE with CO_2_ have been determined, and their effect on the antioxidant, antimicrobial and anticancer activity have been observed. The additional objective of our study was to determine the biological activity of the mixture of both obtained extracts. Antimicrobial activity on the growth of three selected microorganisms and the metabolic activity of metastatic cells were also studied, as well as the crystallinity profile.

The study revealed the highest total phenolic content (1455.37 ± 12.34 mg GAE/100 g) in the mixG extract and the highest antioxidant potential (78.17 ± 0.86%) in the SCG extract. The inhibition of microbial growth of the extract was moderate as the extract successfully inhibited tested microorganisms (*E. coli, S. aureus, C. albicans*) at higher concentrations. The most susceptible tested microorganism was *C. albicans*, where the lowest MIC (40 mg/mL) value was determined. Isolation of a variety of bioactive compounds from ginger with different content depends on many factors, including ginger’s origin, age, and growth conditions, as well as different extraction techniques performed and operating conditions. Furthermore, this significantly impacts the MIC values achieved, which makes an important contribution to making our results differ from the results of many other studies.

All three types of extracts (SCG, UAG and mixG) extract showed excellent anticancer properties. MixG inhibited the metabolic activity of melanoma cells WM-266-4 at a concentration of 0.01 mg/mL by 62% and by 77.5% at a concentration of 0.05 mg/mL. The anticancer activity was significantly influenced by the high content of two main bioactive substances, 6-gingerol and 6-shogaoll, the concentration of which was the highest in the SCG extract (302.49 ± 4.63 mg/g extract of 6-gingerol and 110.96 ± 6.64 mg/g extract of 6-shogaol). The excellent anticancer activity was also significantly influenced by the great antioxidant activity of the obtained extracts, as the anticancer and antioxidant activity are often correlated, while in contrast, the antimicrobial efficacy of the extracts was not well expressed. 

The novelty of this study is the characterization of a mixture of ginger extracts obtained by two different extraction techniques, which can significantly contribute to the greater absorption of bioactive compounds when taken orally due to the amorphous profile.

Based on the obtained results, which show the efficient synergistic effect of polar and nonpolar bioactive compounds in a mixture of two differently obtained extracts, we can confirm that using a mixture of extracts is significant for achieving better biological activities of samples, including excellent absorption of bioactive substances in the human body. It would be advisable to isolate individual bioactive compounds and investigate the biological activity of each compound in the future.

The studies in the literature are mainly based on well-researched organic ginger. Our research has shown that despite some discrepancies in our results compared to the biological activity of organic ginger from different studies, commercial ginger is also a rich source of bioactive substances with good antimicrobial and anticancer properties.

## Figures and Tables

**Table 1:** Content of 6-gingerol and 6-shoagol in all three prepared ginger extracts (UAG, SCG, and mixG extracts) expressed as mg of each compound per g of the respective extract, determined by the LC-MS/MS method.

Sample	6-Gingerol	6-Shogaol
	[mg/g Extract]	[mg/g Extract]
UAG extract	152.72 ± 5.67	60.86 ± 5.85
SCG extract	302.49 ± 4.63	110.96 ± 6.64
mixG extract	270.63 ± 2.04	99.76 ± 0.87

**Table 2:** Antioxidant activity expressed as % inhibition, and content of total phenols expressed as mg of GAE per 100 grams of an individual type of ginger extracts obtained (UAG, SCG, and mixG extracts) determined by appropriate spectrophotometric methods.

Sample	Total Phenolic Content	DPPH
	[mg GAE/100 g]	[% Inhibition]
UAG extract	1342.59 ± 56.75	69.81 ± 1.21
SCG extract	827.38 ± 6.58	78.17 ± 0.86
MixG extract	1455.37 ± 12.34	73.98 ± 0.90

**Table 3:** Determined MIC values for testing the antimicrobial activity of mixG extract on the growth of the tested microorganisms (*E. coli*, *S. aureus* and *C. albicans*) using the microdilution method. MIC values correspond to the minimum extract concentrations that inhibit the growth of microbial cells, expressed in mg/mL.

Extract	Microorganism	MIC [mg/mL]
MixG extract	*S. aureus*	70
	*E. coli*	60
	*C. albicans*	40

## Data Availability

The original contributions presented in the study are included in the article; further inquiries can be directed to the corresponding authors.
